# Cinchonine induces apoptosis of HeLa and A549 cells through targeting TRAF6

**DOI:** 10.1186/s13046-017-0502-8

**Published:** 2017-02-23

**Authors:** Yonghao Qi, Ambara R. Pradipta, Miao Li, Xuan Zhao, Lulu Lu, Xuegang Fu, Jing Wei, Richard P. Hsung, Katsunori Tanaka, Lijun Zhou

**Affiliations:** 1 Tianjin Key Laboratory for Modern Drug Delivery & High-Efficiency, School of Pharmaceutical Science and Technology, Tianjin University, Tianjin, Nankai District 300072 People’s Republic of China; 2 Biofunctional Synthetic Chemistry Laboratory, RIKEN, 2–1 Hirosawa, Saitama, Wako 351-0198 Japan; 3 School of Pharmacy, University of Wisconsin, 777 Highland Avenue, Madison, WI 53705-2222 USA; 4 Biofunctional Chemistry Laboratory, A. Butlerov Institute of Chemistry, Kazan Federal University, 18 Kremlyovskaya street, Kazan, 420008 Russia; 5 JST-PRESTO, 2-1 Hirosawa, Saitama, Wako 351-0198 Japan

**Keywords:** Cinchonine, RING domain of TRAF6, AKT and TAK1 activations and phosphorylations, Immunofluorescence staining, Ubiquitination

## Abstract

**Background:**

Cancer cells are known to over-express TRAF6 that is critical for both AKT and TAK1 activations. The Really Interesting New Gene (RING) domain of TRAF6 is believed to be responsible for the E3 ligase activity, ZINC fingers of TRAF6 provide critical support for the activity of the RING domain which is critical for both AKT and TAK1 activations.

**Methods:**

We employed computational docking program to identify small molecules that could effectively and competitively bind with the RING domain of TRAF6, which is believed to be responsible for its E3 ligase activity. MTT assay and flow cytometry were employed to analyze apoptosis of cancer cells. Signaling pathways were detected using immunoprecipitation and western blotting, and immunofluorescence was pursued to assess the nature of binding of cinchonine to TRAF6. We also performed animal experiments to test effect of cinchonine in vivo.

**Results:**

Cinchonine, a naturally occurring Cinchona alkaloid identified from the docking study, could bind to TRAF6 in HeLa and A549 cells and induce apoptosis of these cancer cells. We found that AKT ubiquitination and phosphorylation as well as phosphorylation of TAK1 were decreased. These activities would lead to subsequent suppression anti-apoptotic protein Bcl-2, while elevating pro-apoptotic protein Bax. Immunofluorescence staining unambiguously demonstrated the binding of cinchonine specifically at the RING domain of TRAF6 in cells, thereby validating the computational modeling. Animal experiments showed that cinchonine could suppress tumor growth in mice without showing significant acute toxicity.

**Conclusion:**

These investigations suggest that through competitive binding with the RING domain of TRAF6, cinchonine could induce apoptosis via inhibiting AKT and TAK1 signaling pathways.

**Electronic supplementary material:**

The online version of this article (doi:10.1186/s13046-017-0502-8) contains supplementary material, which is available to authorized users.

## Background

Tumor necrosis factor (TNF) receptor associated factor 6 (TRAF6) like other TRAF members plays an indispensible role in intracellular signal transductions of an array of receptor families such as T-cell/B-cell receptors and the TNF receptor superfamily [1]. TRAF6 acts as a direct E3 ligase for protein kinase B (AKT) and can also activate transforming growth factor activated kinase 1 (TAK1) [2, 3]. More significantly, it is also over-expressed in cancer cells [4–9]. Structurally, TRAF6 consists of four parts: the Really Interesting New Gene (RING) domain, ZINC finger domain, a coiled-coil domain, and a C-terminal TRAF-C domain [10]. While the RING domain of TRAF6 is believed to function as an E3 ubiquitin ligase, ZINC fingers of TRAF6 provide critical support for the E3 ligase activity of the RING domain [10–12]. Binding of the RING domain of TRAF6 with ubiquitin-conjugating enzyme (Ubc13) and ubiquitin-conjugating enzyme variant (UEV1A) is believed to be crucial for the Lys-63 dependent activation of both AKT [2] and TAK1 [3, 13, 14]. In recently studies, many researchers have found that the level of AKT phosphorylations at Thr-308 and Ser-473 were significantly reduced in TRAF6−/− mouse embryonic fibroblasts relative to TRAF6+/+ [2]. In addition, it was reported that in mouse myoblasts, knockdown of TRAF6 appears to compromise both TAK1 and AKT signaling pathways [15].

Both AKT and TAK1 are involved in growth factors, metabolism, cell proliferation, survival, apoptosis and inflammatory responses [16–19]. Furthermore, AKT and TAK1 can also accelerate the activation of downstream nuclear factor κB (NF-κB) via phosphorylation of inhibitor of NF-κB and then regulate apoptosis-related kinases Bax/Bcl-2 [20–23], activator protein-1 and p38/mitogen-activated protein kinase signaling pathways [24–27]. Previously in our own studies, we have uncovered that a small molecule could bind at the RING domain of TRAF6, leading to inhibition of the AKT activity [28]. Considering the strong association between TRAF6 and activations of both AKT and TAK1 pathways, and their implications on apoptosis and cell proliferation as well as a possible therapeutic approach for treatment of cancer, we employed computational docking to identify small molecules that can specifically bind with the RING domain of TRAF6 and could compete with the binding of its natural ligand Ubc13. We wish to report herein our studies designed to explore the mechanism of which a small molecule could block activations of AKT and TAK1 and subsequently induce apoptosis of cancer cells in vivo and in vitro.

## Methods

### Materials

HeLa and A549 cells were provided from Tianjin International joint Academy of Biomedicine, Normal human dermal fibroblast (NHDF) cells were provided from Professor Jun Dai (Tianjin University, China). RPMI-1640, DMEM, and FBS were purchased from Corning, Australia. Cinchonine (Xiensi Biochemical Technology Co, Tianjin, China) was dissolved in DMSO (Sigma-Aldrich). Phosphatase inhibitor and 3-(4,5-dimethyl-2-thiazoyl)-2,5-diphenyl-2H-tetrazolium bromide (MTT) were obtained from Sigma, USA. HEPES was purchased from Solarbio (Beijing, China). Apoptosis Detection Kit was purchased from Becton-Dickinson, USA. Polyvinylidene Fluoride (PVDF) membrane was purchased from Merk Millipore, USA. In Situ Apoptosis Detection Kit, POD was purchased from BOSTER (Wuhan, China). Protein A Sepharose beads were brought from Pierce, USA. Balb/c-nude mice and Kunming mice were purchased from Yi Sheng Yuan Bio Logical Technology Co. Tianjin, China.

Antibodies used in this study include the following: Ubiquitination antibody and rabbit anti-TRAF6 polyclonal antibody (Santa Cruz, USA); Rabbit polyclonal antibody against total AKT, phosphorylation-AKT (Thr-308), phosphorylation-AKT (Ser-473), total TAK1, phosphorylation-TAK1 (Thr-184/Thr-187), Bax, and Bcl-2 (Cell Signaling Technology, USA); Rabbit polyclonal antibody against TRAF6, ALEXA FLUOR 647 conjugated (Bioss Inc. USA); Rabbit anti β-actin polyclonal antibody, Goat anti-mouse, and goat anti-rabbit IgG polyclonal antibody (Beijing Zhongshan Golden Bridge Biotechnology Co., Ltd, China).

### Cell culture

HeLa and A549 cells were cultured in RPMI-1640, while NHDF cells were cultured in DMEM, both media were supplemented with 10% FBS. All cells were incubated at 37 °C in a humidified atmosphere of 5% CO_2_.

### Docking study

To first identify small molecules that could bind with the RING domain of TRAF6, the three-dimensional structure of the RING and ZINC finger domains (residues 50–159) of TRAF6 was obtained from the Protein Data Bank database with an access code of 3HCT [29]. The structure was used in the docking experiments, and the docking grid was placed over the RING domain (residues 67–124) of TRAF6, which is believed to interact with Ubc13. We carried out computational screening of a small library of chemical compounds established by Jkchemical Sigma, and Alfa Aesar that would include 1792 commercially available compounds. All structures were built using a 2D/3D editor-sketcher and were minimized to a local energy minimum using the CHARMm-like force field. This library was docked into TRAF6 implemented by AutoDock4.10 software. Compounds were filtered by the predicted binding free energy. Further filtering rules were guided by chemical diversity, rigidity, novelty and actual commercial availability.

### 3-(4, 5-Dimethylthiazol-2-yl)-2, 5-diphenyltetrazolium bromide (MTT) assay

Cells were seeded into 96-well plate at density of 4.1 × 10^3^ cells/well. Different concentrations (1, 5, 10, 50, 100, and 250 μM) of cinchonine were added to these wells. Cis-platinum (16.7 μM) was used as a positive control. After treating with cinchonine for 48, 72, and 96 h respectively, the medium with different concentrations were removed and cells were incubated with 20 μL MTT (5 mg/mL) for 4 h at 37 °C. The MTT solution was discarded and formazan was dissolved in 150 μL DMSO. The absorbance of each well was read using a Microplate reader (Shanghai Kehua, China) at 490 nm [30]. Cell inhibition ratio was calculated using the equation:

$$ \mathrm{Cell}\kern0.5em \mathrm{inibition}\kern0.5em \mathrm{ratio}\kern0.5em \left(\%\right)=\left(1\hbox{-} \mathrm{ODtreated}/\mathrm{ODcontrol}\right)\times 100\% $$

Half maximal inhibitory concentration (IC_50_) was calculated by regression curve.

### Flow cytometry

Apoptosis of the cells were determined using the Annexin V-FITC Apoptosis Detection Kit (Becton-Dickinson, Franklin Lakes, NJ, USA) according to the manufacturer’s protocol [30]. In brief, cells were seeded into 60-mm plate and the confluence was allowed to reach 50%–60%. After which, according to MTT result, cinchonine was added at concentrations of 100 μM, 150 μM, and 180 μM for HeLa cells, and at concentrations of 150 μM, 180 μM, and 220 μM for A549 cells at 37 °C for 48 h. Whereafter, cells were washed twice in cold PBS and then resuspended in 1× binging buffer, and 100 μL of the cell suspension was transferred to a 5 mL culture tube, to which 5 μL Annexin V-FITC and PI were added. The mixture was incubated for 15 min at room temperature in the dark. Results were immediately analyzed with a FACScalibur flow cytometer and cell Quest Pro 5.1 (BD, Biosciences, Franklin Lakes, NJ, USA).

### Immunoprecipitation

Cells were extracted with lysis buffer containing 50 mM HEPES (pH 7.4), 150 mM NaCl, 1% NP-40. The lysate was centrifuged at 12000 × g for 20 min at 4 °C, and the supernatant containing total protein was harvested. Subsequently, the total protein was exposed to anti-body against AKT, which was immobilized on protein A beads (Pierce). The resulting beads were incubated at 4 °C overnight with gentle rotation, washed 3 times with lysis buffer, and separated at 800 × g for 3 min. The desired protein bands were visualized by Western blotting [31].

### Western blot analysis

When the confluence reached 60%–70%, cells were treated with 180 μM of cinchonine for different time. After which, cells were treated with 20 μg/mL of lipopolysaccharide (LPS) for 3 h to induce activation of AKT [31]. To induce activation of TAK1, 1 μg/mL of LPS was used to treat the cells for 15 min [32]. The protein were extracted with lysis buffer for 30 min on ice. Extracts were centrifuged at 12000 × g for 20 min at 4 °C, and the supernatants containing total protein were harvested. Each sample containing 50 μg protein was separated by 10% SDS-PAGE and transferred to PVDF membranes. The membrane was blocked in 5% non-fat milk and incubated overnight at 4 °C with anti-bodies against AKT (1:1000), phosphorylation-AKT (1:1000), TAK1 (1:1000), phosphorylation-TAK1 (1:1000), ubiquitin (1:1000), Bax (1:1000), Bcl-2 (1:1000), β-actin (1:1000). Then the membranes were incubated at room temperature for 1 h with their corresponding secondary anti-bodies. The membranes were incubated with ECL solution (CWBIO) and exposed to the film (FUJI, Japan). The membranes were stripped and reblotted with β-actin antibody to verify the equal loading of protein in each lane. Image J software v.1.48u (National Institutes of Health, Bethesda, MD, USA) was used to quantify the intensity of protein bands.

### Animal experiment

Animals were maintained in Institute of Radiation Medicine Chinese Academy of Medical Science. All experimental procedures using these mice were in accordance with requirements and guidelines for treating experimental animals approved by the National Institutes for Food and Drug Control of China. All animal experiments have been approved by The Ethics Committee on animal experiments of Institute of Radiation Medicine Chinese Academy of Medical Science (The approval number is: (2016) 1–008). Balb/c-nude mice (four weeks old and gender random) were first subcutaneously inoculated with cancer cells (5 × 10^6^ cell/mL) to establish transplanted mode of cervical cancer. After feeding the nude mice for 3 weeks post implantation, the tumor volume was approximately 586.84 mm^3^ when intratumorally injection commenced. The inoculated mice were then divided into: experimental group-I to be intratumorally injected with cinchonine (180 μM), experimental group-II to be intratumorally injected with cinchonine (360 μM), and control group (treated with medium). The injection volume is 100 μL. Dosage of cinchonine administrated to nude mice are 0.265 mg/Kg (180 μM) and 0.530 mg/Kg (360 μM). The weight of nude mice and the shortest and longest diameter of tumor were measured with calipers with an interval of every other day. Tumor volume (mm^3^) was calculated using the following standard formula: (the longest diameter) × (the shortest diameter)^2^/2. The hyperplasia rate was calculated using: (“the volume of nude mice in day 2, 4, 6, 8, 10, 12, and 14” – “the volume of nude mice in day 2”)/“the volume of nude mice in day 2”. There were 5 nude mice in each group.

For the acute toxicity test, we used high-dosage intravenous injection, 500 μL of cinchonine (360 μM) with injected into 5 male and 5 female Kunming mice, and injected mice were observed for 14 d.

After treating with cinchonine for 14 d, paraffin sections were prepared from tumor tissues in the following manner. The resulting paraffin sections were then treated with an Apoptosis Detection kit (BOSTER, Wuhan, China) according to the manufacturer's protocol [33]. Briefly, paraffin sections were first incubated in electric dry oven at 60 °C for 30 min before they were placed into dimethyl benzene for 10 min and in fresh dimethyl benzene for another 10 min. Subsequently, these section were placed in a gradient of ethanol (5 min each ethanol absolute, 95 and 75% ethanol). The proteinase K was used to incubate them for 15 min at room temperature before being washed with 0.01 M phosphate buffer saline solution. After these paraffin sections were cultivated in terminal deoxynucleotidyl transferase (TdT) buffer containing TdT and biotinylated DNA uracil nucleoside triphosphate (dUTP) in TdT buffer, they were incubated in a moist atmosphere at 37 °C for 2 h and washed with TBS. Paraffin sections were then sealed and cultivated with biotin in a wet atmosphere for 30 min at room temperature before being washed with TBS. These sections were then incubated with Strept Avidin-Biotin Complex (SABC) for 30 min in 37 °C and washed with TBS. Lastly, paraffin sections were dyed with diaminobenzidine (DAB) and counterstained with Hematoxylin [34, 35], and the final toxicity analysis was viewed under an optical microscope.

### Immunofluorescence staining

The fluorescent probe, 7-(N,N-diethylamino) coumarin-N-(4-bromobenzyl)-3-carboxamide, was conjugated to the terminal olefin of cinchonine according to the following procedure. A mixture of 7-aminocoumarin (150.0 mg, 0.35 mmol), cinchonine (51.4 mg, 0.18 mmol), Pd(OAc)_2_ (2.00 mg, 8.7 μmol), PPh_3_ (4.80 mg, 18.0 μmol) and Et_3_N (49.0 μL, 0.40 mmol) in anhydrous toluene (2 mL) was capped under N_2_ atmosphere and heated at 110 °C for 24 h [36]. The resulting mixture was then allowed to cool to room temperature, filtered through Celite^TM^, and washed with CHCl_3_. The filtrate was concentrated to dryness under reduced pressure, and the resulting crude was purified using reversed-phase HPLC to give the desired coumarin probe conjugated cinchonine as yellow solid (17.0 mg, 15%).

Conditions for reversed-phase HPLC: Column, Cosmosil 5C18-AR300 (Nacalai Tesque, Inc.) 10 × 250 mm; Mobile phase A, 0.1% TFA in H_2_O; B, 0.1% TFA in CH3CN; Gradient elution, 0–4 min at 5% B, 4–34 min at 5–95% B, 34–35 min at 95% B; Flow rate at 4 mL/min; UV detection at 254 nm. 1H NMR (400 MHz, CDCl_3_, 25 °C) δ 9.42 (t, J = 5.8 Hz, 1H, NH), 9.10 (d, J = 5.5 Hz, 1H), 8.70 (s, 1H), 8.40 (dd, J = 14.8, 8.6 Hz, 2H), 8.26 (d, J = 5.4 Hz, 1H), 7.99 (t, J = 7.7 Hz, 1H), 7.84 (t, J = 7.7 Hz, 1H), 7.44 (d, J = 9.0 Hz, 1H), 7.34 (q, J = 9.5 Hz, 4H), 6.67 (dd, J = 9.0, 2.4 Hz, 1H), 6.59 (s, 1H), 6.53 (d, J = 15.8 Hz, 1H), 6.49 (d, J = 2.3 Hz, 1H), 6.33 (dd, J = 15.8, 7.7 Hz, 1H), 4.63 (d, J = 5.6 Hz, 2H), 4.40 (dd, J = 14.0, 7.1 Hz, 1H), 3.64–3.62 (m, 1H), 3.50–3.44 (m, 6H), 3.27 (q, J = 10.3 Hz, 1H), 2.79 (q, J = 8.2 Hz, 1H), 2.48 (t, J = 11.6 Hz, 1H), 2.14 (s, 1H), 2.02 (t, J = 8.4 Hz, 1H), 1.82 (q, J = 10.5 Hz, 1H), 1.24 (t, J = 7.1 Hz, 6H), 1.15–1.08 (m, 1H); ESI-HRMS m/z calcd for C_40_H_43_N_4_O_4_ ([M + H]+) 643.3279, found 643.3282.

Cells grown on glass coverslips were incubated with the fluorescent probe conjugated cinchonine for 1 h and washed with ice-cold PBS. These cells were subsequently fixed in 95% ethanol at room temperature for 30 min and permeabilized with 0.2% Triton X-100. After being blocked with 5% BSA in PBS, the cells were incubated with Rabbit polyclonal antibody against TRAF6, ALEXA FLUOR 647 conjugated (Bioss Inc. USA) for 2 h at 37 °C [37]. Co-localization of modified cinchonine and TRAF6 was analyzed using fluorescence microscope. The fluorescence microscope used for immunofluorescence observation is Nikon eclipse 80i and the light is Nikon INTENSILIGHT C-HGFI. The analysis software is NIS-Elements. The image is a single layer. There was no quantitative analysis of the co-localization.

### Statistical analysis

Statistical analysis was performed with SPSS software and the experimental values which between difference groups were shown as the mean ± standard deviations. The statistical significance of differences between the control and treated groups were determined by Student’s *t*-test. Values *p < 0.05 and **p < 0.01 are considered significant.

## Results

### Docking study and identification of cinchonine

**Fig. 1:**
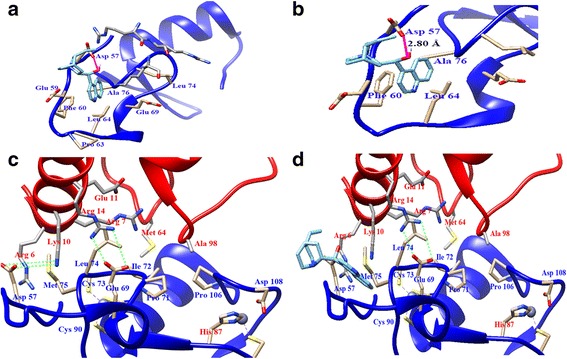
Docking result of cinchonine. **a** Binding of cinchonine (*in cyan*) with TRAF6. **b** The -OH group of cinchonine forges one hydrogen bond (*in pink*) with residues Asp57 in TRAF6. **c** The binding of Ubc13 (*in red*) to TRAF6 (*in blue*). **d** The influence of cinchonine on the interaction between TRAF6 and Ubc13

We found cinchonine (Additional file 1: Figure S1), a member of Cinchona alkaloid family, to be the most effective in binding to the RING domain of TRAF6. More specifically, we found four possible areas for binding of cinchonine to the RING domain. As shown in Fig. 1a, after analyzing all four possible areas and comparing their free energies, the most suitable binding mode for cinchonine was determined, as it possesses the lowest free energy (△G = −6.64 kcal/mol) of the four areas analyzed. In this binding mode, cinchonine appears to be surrounded by amino acid residues of Asp-57, Glu-59, Phe-60, Pro-63, Leu-64, Leu-74, and Ala-76, thereby revealing also significant interactions with residues preceding (54–66) the RING domain (67–124). From the expanded view as shown in Fig. 1b, there appear to be hydrophobic interactions between the quinoline motif of cinchonine and residues Phe-60, Leu-64, and Ala-76 of TRAF6. More significantly, we were able to verify that the hydroxy group (−OH) of cinchonine forges a key hydrogen bond with Asp-57, whereas the terminal olefin of cinchonine does not interact with amino acid residues of TRAF6. These structural observations provide a critical basis to pursue possible visualization of such interaction in cells using a synthetically modified cinchonine that would be substituted with a fluorescent probe. In particular, we envisioned that if an appropriate fluorophore could be conjugated at the terminal olefin of cinchonine, it would remain outside the RING domain and would not interfere the binding of cinchonine at this region.

### Inhibition of proliferation and induction of early apoptosis in HeLa and A549 cells

**Fig. 2:**
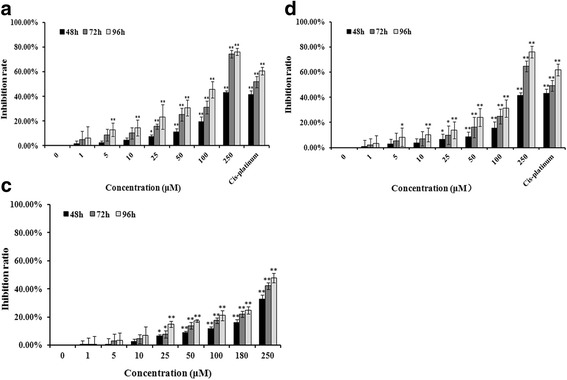
Assessment of apoptosis by MTT assay. **a** The inhibition ratio of HeLa cells after treated with cinchonine for 48, 72, and 96 h. **b** and **c** represented A549 and NHDF cells. Quantitative analysis of MTT represented as the mean ± SD of three independent experiments. *P < 0.05 and **P < 0.01 when compared to untreated control

Western blot assay showed that HeLa and A549 cells established high expression levels of TRAF6 (Additional file 1: Figure S2). In Figs. 2a and b, cinchonine inhibited HeLa and A549 cells proliferation in a dose- and time-dependent manner. In NHDF cells, using the same concentration gradient, the inhibition ratio was significantly lower than HeLa and A549 cells (Fig. 2c). With the concentration of cinchonine being set at 180 μM, inhibition rates were 16.08, 22.07 and 24.95%, respectively, for durations of 48, 72, and 96 h. We subsequently calculated the half maximal (50%) inhibitory concentration (IC_50_) value to be 180 μM through the computer-estimated using the Pharmacologic Calculation Program [38] and employed this concentration for the following experiments.

**Fig. 3:**
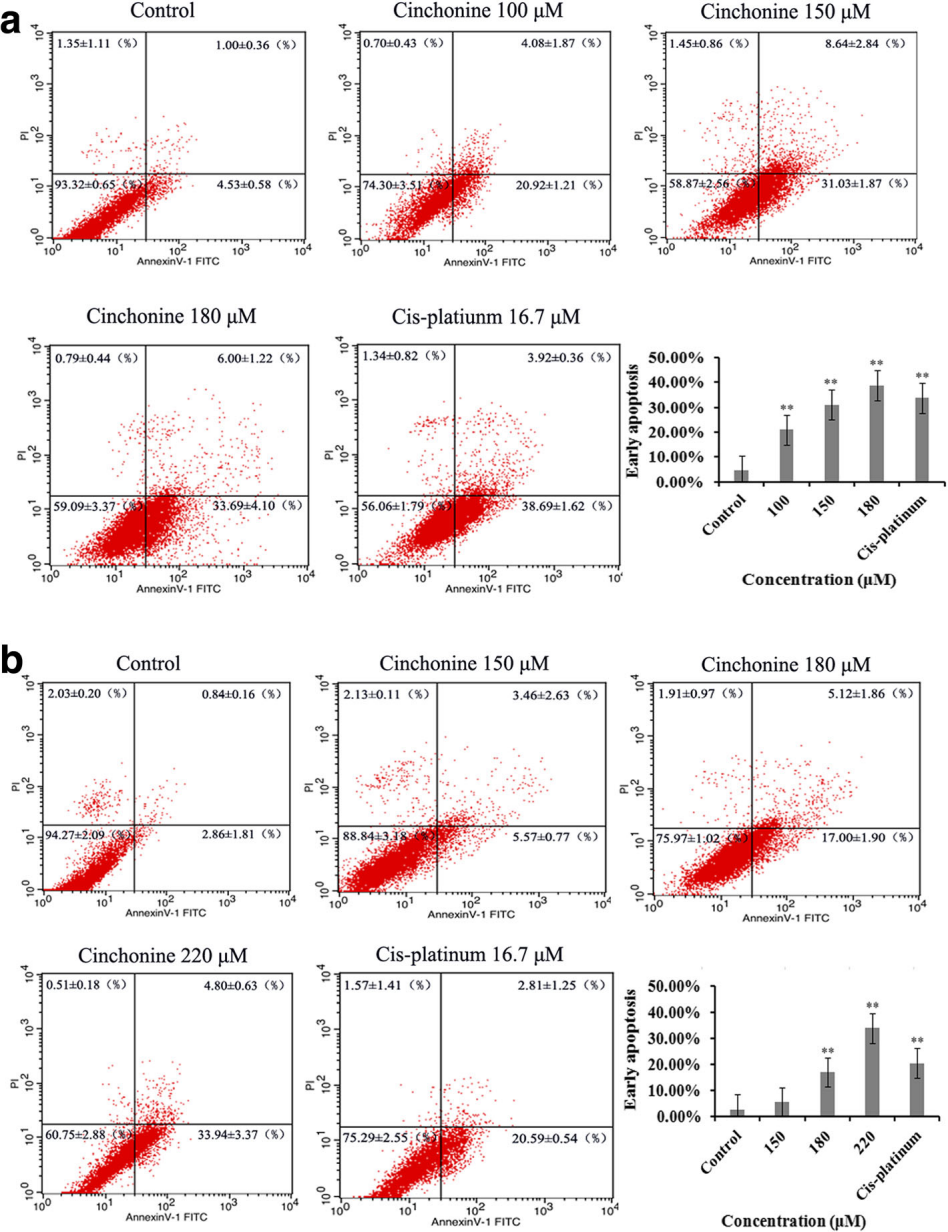
Cinchonine induces the early apoptosis. The apoptosis level of HeLa (**a**) and A549 (**b**) cells were measured using the Annexin V-FITC and Propidium iodide assay at 48 h after treatment with cinchonine. The results show cinchonine induces early apoptosis of cells in a concentration-dependent manner. Cis-platinum (16.7 μM) was used as a positive control. Quantitative analysis of flow cytometry results represented as the mean ± SD of three independent experiments. *P < 0.05 and **P < 0.01 when compared to control group

After treatment with cinchonine for 48 h, the percent of early apoptosis in control group was 4.53% ± 0.58. The percent of apoptosis increased after being treated with cinchonine. The fraction of early apoptosis increased to 20.92% ± 1.21 when treated with 100 μM of cinchonine and to 31.03% ± 1.87 with 150 μM of cinchonine, while reaching 38.69% ± 1.62 with 180 μM of cinchonine. As a positive control, Cis-platinum (16.7 μM) showed 33.69% ± 4.10 of cells in the state of early apoptosis (Fig. 3a). Therefore, we have demonstrated here that cinchonine could induce early apoptosis in HeLa cells as well as in A549 cells (Fig. 3b).

### Changes in AKT and TAK1 activations and levels of Bax/Bcl-2

**Fig. 4:**
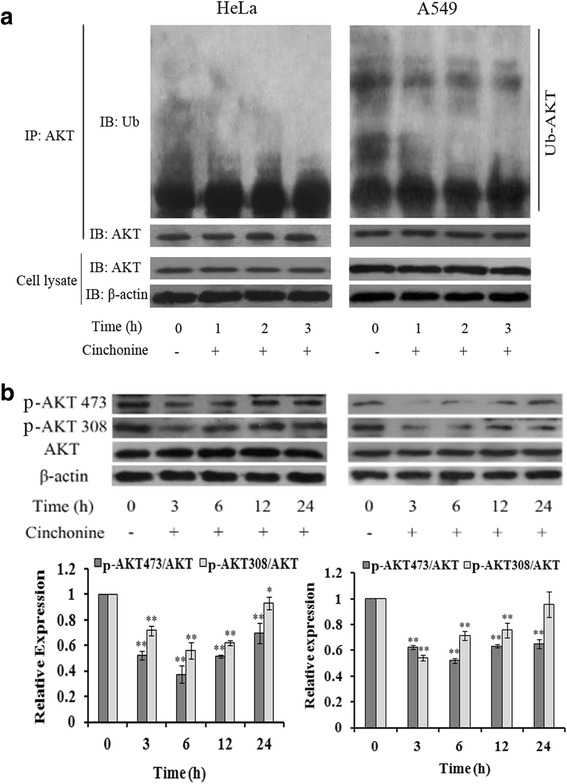
Cinchonine inhibited AKT activation. **a** Cells were exposed to cinchonine (180 μM) for 0, 3, 6, 12, and 24 h. Immunoprecipitation was performed to determine the expression level of ubiquitination of AKT. Result shows that the ubiquitination-AKT was lower with the treatment of cinchonine. **b** Cells were pre-treated with cinchonine for the indicated durations and Western blotting was carried out to show us that cinchonine down-regulated the phosphorylation of AKT significantly at 3 and 6 h. The results shown are the mean ± SD of three independent experiments. *P < 0.05 and **P < 0.01

**Fig. 5:**
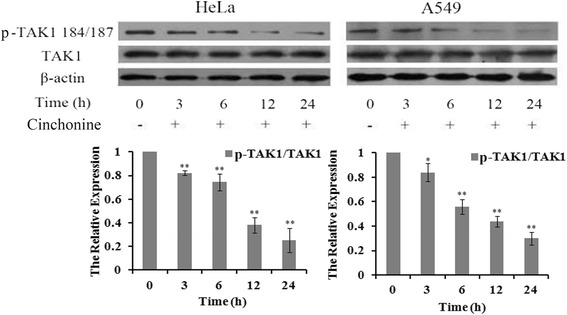
Cinchonine inhibited phosphorylation of TAK1. Cells were treated with cinchonine (180 μM) for 0, 3, 6,12, and 24 h. Western blotting was performed to determine the expression level of phosphorylation of TAK1. Result shows that cinchonine inhibited phosphorylation of TAK1. The results shown are the mean ± SD of three independent experiments. *P < 0.05 and **P < 0.01

**Fig. 6:**
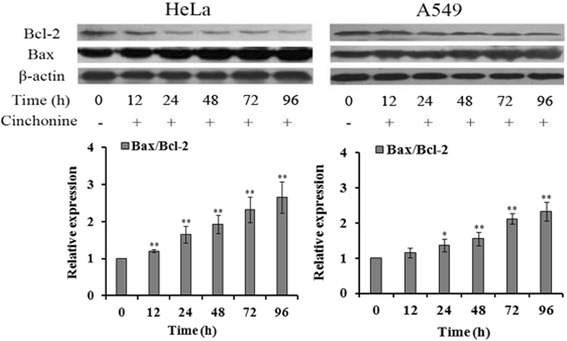
Changes of the expression levels of Bax/Bcl-2. Cells were treated with cinchonine (180 μM) for the indicated durations. Western blotting was subsequently performed to determine the expression of Bax/Bcl-2 in the cell lines. Result shows that the expression level of Bax was increased while Bcl-2 was decreased. The data represent mean ± SD of three independent experiments. *P < 0.05 and **P < 0.01

To understand the underlying molecular mechanism of cinchonine induced apoptosis, ubiquitination and phosphorylation levels of AKT were analyzed in these two cell lines. Immunoprecipitation showed that ubiquitination of AKT was down-regulated after being treated with cinchonine for 1, 2 and 3 h (Fig. 4a). Furthermore, in Fig. 4b, phosphorylations of AKT at Thr-308 and Ser-473 were significantly impeded after treatment for 3 and 6 h. Phosphorylation levels of TAK1 at Thr-184 and Thr-187 were analyzed and found reduced after exposing these two cell lines to cinchonine (Fig. 5). Levels of downstream apoptosis-related kinases protein Bax (pro-apoptotic protein)) and Bcl-2 (anti-apoptotic protein) were also examined. After cells were treated with cinchonine for 12, 24, 48, 72 and 96 h, the expression level of Bax was increased significantly, while the level of Bcl-2 decreased in a time-dependent manner in both cell lines (Fig. 6). These results provide tangible evidence that cinchonine could influence both AKT and TAK1 signaling pathways as well as their downstream proteins like though its binding with TRAF6.

### Possible tumor suppression in vivo

**Fig. 7:**
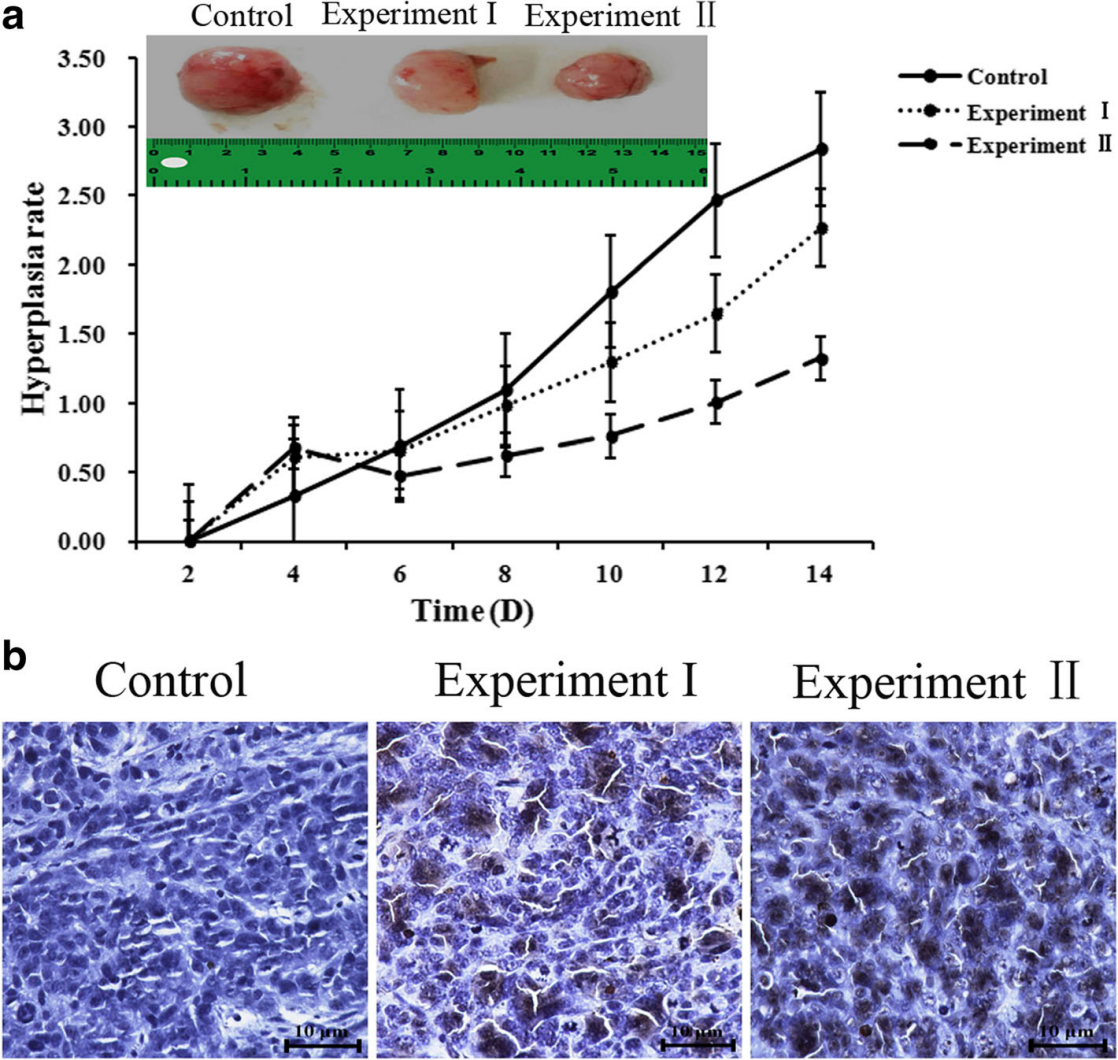
Cinchonine inhibited tumor growth and induced apoptosis in nude mice. **a** The growth ratio of the control group (2.839 ± 0.583) was greater than experiment I (2.266 ± 0.467) and experiment II (1.324 ± 0.367 *P* < 0.05). **b** The control group, which was treated with no cinchonine, showed nearly no TUNEL positive cells. In experimental group-I and group-II, showed more TUNEL positive cells when treated with cinchonine. The result demonstrates that cinchonine can induce apoptosis and the quantity of apoptosis is dose-dependent

Tumor model in nude mice was successfully established, and the tumorigenic rate was 100%. Uptake of food and drink of nude mice was normal, and the mental state was good. The tumor growth ratio of the experiment group-I was 2.267 ± 0.467, while the experiment group-II was 1.324 ± 0.367, which is significantly lower than the control group: 2.839 ± 0.583 (Fig. 7a). Weights of these mice were approximately 18–22 g (Additiona﻿l file 2: Table S1). Uptake of food and drink of all Kunming mice was normal and the mental state was good without any death or loss of mobility after treatment with cinchonine for 14 d.

Comparing tumor tissues of nude mice from the three groups, in situ TUNEL assay shows that experimental groups have more nuclei being dyed than that of the control group, thereby revealing the ability of cinchonine to induce apoptosis of transplanted tumor cells in nude mice (Fig. 7b).

### Visualizing the binding of cinchonine with TRFA6 in HeLa and A549 cells

**Fig. 8:**
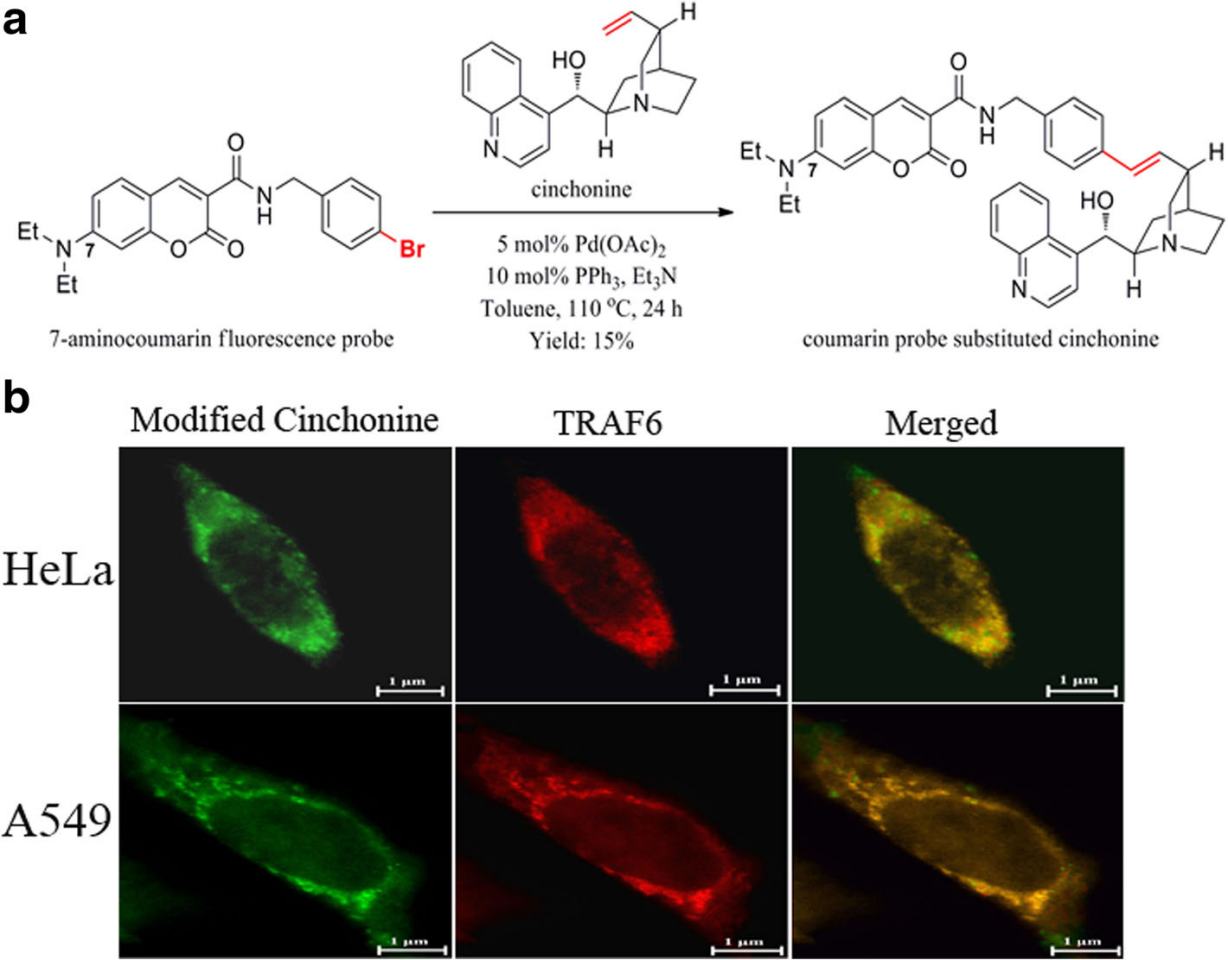
Visualizing binding of cinchonine with TRAF6 in HeLa and A549 cells. **a** Synthesis of a fluorescent probe conjugated cinchonine. **b** Immunofluorescence staining images of the localizations of TRAF6 and cinchonine in cells. Modified cinchonine (*green fluorescence*) and TRAF6 (*red fluorescence*) were co-located to cytoplasm

Results of our docking study suggest that cinchonine could effectively bind with TRAF6. To visualize such interaction in cells, a coumarin-derived fluorescent probe was conjugated specifically to the terminal olefin of cinchonine via synthesis (Fig. 8a). Immunofluorescence staining experiment using this modified cinchonine was performed. Modified cinchonine (green fluorescence) and TRAF6 (red fluorescence) were seen co-located in the cytoplasm of HeLa and A549 cells (Fig. 8b), thereby supporting the assessment that cinchonine could bind with TRAF6 in cells.

## Discussion

Wu et al. found that mutations of residues Asp-57 (D57K), Ile-72 (I72D), and Leu-74 (L74E or L74K) in TRAF6 had significantly disrupted the TRAF6-Ubc13 interaction, the RING domain accounts for two of these interactions: (a) Folding of residues Ile-72 and Leu-74 within the domain in a manner that they provide a hydrophobic surface for Ubc13 to interact; and (b) a salt bridge between Glu-69 of the RING domain and Arg-14 of Ubc13. The third key interaction involves residues (residues 54–66), salt bridges are also observed in the X-ray crystal structure between Asp-57 and Lys-10/Arg-6 of Ubc13 (Fig. 1c) [29]. Based on these assessments, our docking studies would suggest that the presence of cinchonine, and in particular, the H-bond between its OH group and the amino acid residue Asp-57, could effectively prevent the salt-bridge formation between Asp-57 of TRAF6 and Lys-10/Arg-6 of Ubc13 (Fig. 1d). Consequently, in the absence of this salt-bridge, the formation of the TRAF6-Ubc13 complex would suffer. It is noteworthy that the other salt bridge between Glu-69 of the RING domain and Arg-14 of Ubc13 appears to remain intact and unaffected by cinchonine (Fig. 1d). However, without the assistance of a second salt bridge, it would be difficult for Ubc13 to anchor firmly on the surface of the RING domain of TRAF6, or at least, Ubc-13 would be unable to complex properly with TRAF6 in a manner sufficient for the ensuing activation of AKT and TAK1.

The main purpose of this work is to explore whether the RING domain in TRFA6 could serve as a viable anti-cancer therapeutic target. Because HeLa and A549 cells have a high expression level of TRAF6, we chose HeLa and A549 cells. The expression level of TRAF6 was shown in Supporting Information (Additional file 1: Figure S2).

Armed with these detailed computational analysis and Western blotting results, we proceeded to pursue cell-based assays using HeLa, A549, and NHDF (as controls) cells, and found that cinchonine could inhibit proliferations of HeLa and A549 cells in a concentration- and time-dependent manner. NHDF cells were used to test cytotoxicity of cinchonine. The experiment showed that cinchonine exhibits low cytotoxicity when used at low concentrations. When the concentration of cinchonine is set at 180 μM, the percent inhibition of NHDF cells was also much lower than those of HeLa and A549 cells. Karbwang et al. had reported pharmacokinetics of quinine, quinidine, and cinchonine when given as a combination to patients with falciparum malaria. This combination was given intravenously to patients every 8 h either at 400 mg or 600 mg dosage containing one third of each component. The maximum amount of cinchonine used in each dosage would reach as high as 200 mg, which is 170 μM [39] and close to the IC50 value (180 μM) of cinchonine in our study. Therefore, we chose 180 μM as an optimal concentration to conduct subsequent study of mechanism.

Flow cytometry showed that cinchonine could induce early apoptosis while does not cause cell death after treating with cinchonine for 48 h.

AKT is known to be associated with many proteins that contribute to cell survival [32]. Over-expression of TRAF6 could increase phosphorylations of AKT at Thr-308 and Ser-473 as well as AKT ubiquitination, leading to enhanced AKT activity, and many reports suggest that the expression level of ubiquitination-AKT is higher in cancer cells than normal cells [40–42]. It is noteworthy that Yang et al. had found that upon IGF-1 treatment, endogenous AKT ubiquitination was less in TRAF6−/− comparing to that in TRAF6+/+ in primary mouse embryonic [2]. Reduction of ubiquitination-AKT in vivo down-regulated the level of phosphorylation-AKT at Thr-308 and Ser-473 [43, 44]. Lamothe et al. showed that when down-regulating the expression of TRAF6, levels of phosphorylations of AKT at Thr-308 and Ser-473 were lowered [12]. Our results also revealed that cinchonine led to reduced expression levels in both ubiquitination and phosphorylation of AKT, thereby impeding the AKT activation (Figs. 4a and b).

TRAF6 is also known to facilitate phosphorylation of TAK1 and its subsequent activities thereof. Yamashita et al. reported that in TRAF6-deficient mouse embryonic fibroblasts, TAK1 failed to be auto phosphorylated at Thr-184 and Thr-187 and could not activate c-Jun N-terminal kinase (JNK) [3, 27, 45]. Therefore, auto phosphorylation levels of TAK1 were analyzed in these two cell lines and the observed reduction of TAK1 auto phosphorylations is likely associated with the binding of cinchonine to the RING domain of TRAF6.

We had demonstrated that cinchonine could reduce proliferation and induce apoptosis in both HeLa and A549 cells. There are reports documenting that phosphorylation of AKT and TAK1 can inactivate Bax, while increasing the Bcl-2 activity [20–22, 27]. The Bcl-2 protein family contains pro-apoptotic member Bax and anti-apoptotic member Bcl-2, which are considered to be active effectors and regulators [46]. More specifically, Bcl-2 regulates signal pathways that can ensure cell survival, while Bax is a negative regulator of Bcl-2 [47–49]. Linseman et al. had also found that mitochondria play a key role in cell apoptosis because members of the Bcl-2 family proteins can interact with the mitochondrial outer membrane [50], and that Bcl-2 and Bax can directly influence the mitochondrial membrane permeability, caspases, and apoptosis [51, 52]. Based on these literature assessments, we examined expression levels of Bcl-2 and Bax and found that they changed in an expected manner after exposing cells to cinchonine at different time periods.

We conducted preliminary animal studies to explore possible tumor suppression using cinchonine. Some incision enzyme of DNA will be active to make DNA fragmentation when cells undergo apoptosis. TUNEL is a common method for detecting DNA fragmentation in situ [34]. Cinchonine inhibited tumor growth as determined by tumor volume. TUNEL results demonstrated that incision enzyme of DNA was fragmented when treated with cinchonine, thereby confirming that cinchonine could induce apoptosis in nude mice.

In relations to the immune system, reports have shown that the C-terminus (333–508) of TRAF6 binds to CD40, and thus, TRAF6 could play a significant role in immunology [53]. In this study, we chose the RING domain of TRAF6, because it is the binding domain for Ubc13 and represents a potential therapeutic target for cancers. Nevertheless, it is important to recognize that cellular functions of Ubc13 are critical in the context of innate immune responses. By employing methods for targeted gene ablation in mice, Fukushima et al. demonstrated that ablation of the gene encoding Ubc13 in homozygous mice resulted in embryonic lethality. However, despite a reduced level of the Ubc13 protein, heterozygous mice have normal phenotypes and exhibit no abnormalities in immune cell populations [54]. Therefore, these reports suggest that a reduced level of the Ubc13 protein does not appear to compromise functions of the immune system.

Lastly, to ensure that cinchonine could indeed bind with TRAF6 in cells, we designed and synthesized a new cinchonine derivative containing a coumarin-derived fluorescent probe. It is important to note that the 7-aminocoumarin probe was specifically conjugated to the terminal olefin of cinchonine because the resulting fluorophore should remain outside the RING domain based on the modeling. Thus, such structural modification would not diminish the ability of cinchonine to interact with TRAF6. Subsequent immunofluorescence staining distinctly verifies that cinchonine could bind with TRAF6 in cells, and thus, it supports our docking studies and further substantiates the potential of TRAF6 serving as an antitumor target.

## Conclusions

In summary, computational modeling predicted that cinchonine could complex with the RING domain of TRAF6, leading to disruption of the binding with its natural ligand the Ubc13 protein that is critical for initiating downstream events of AKT and TAK1 activations. Immmunofluorescence staining validates such interaction in cells when using modified cinchonine containing a coumarin-derived fluorescent probe. Subsequent both in vitro and in vivo experiments distinctly demonstrate that cinchonine could induce apoptosis and reduce proliferation of cancer cells; and that the mode of action is blocking the AKT and TAK1 activations though interfering the formation of the TRAF6-Ubc13 complex. The RING domain of TRFA6 can be regarded as a potentially useful antitumor target and efforts are underway to further investigate such potential.

## Additional files

Additional file 1:
**Figure S1.** Chemical structures of cinchonine and probe substituted cinchonine. **Figure S2.** The expression level of TRAF6. (DOC 80 kb)
Additional file 2: Table S1. Weight of the nude mice. (DOCX 15 kb)

## Abbreviations

AKT: Protein kinase B
AP-1: Activator protein-1
Bax: B-cell lymphoma-2 associated X protein
Bcl-2: B-cell lymphoma leuemia-2
DAB: Diaminobenzidine
FITC: Fluorescein isothiocyanate
IKK: Inhibitor of nuclear factor kappa-B
JNK: c-Jun N-terminal kinase
LPS: Lipopolysaccharide
NF-κB: Nuclear factor κB
PI: Propidium iodide
PVDF: Polyvinylidene Fluoride
RING: Really Interesting New Gene
SDS-PAGE: Sodium dodecyl sulfate polyacrylamide gel electrophoresis
TAK1: Transforming growth factor activated kinase 1
TNF: Tumor necrosis factor
TRAF6: Tumor necrosis factor receptor associated factor 6
TUNEL: Transferase UTP nick end labeling
Ubc13: Ubiquitin-conjugating enzyme E2
UEV1A: Ubiquitin-conjugating enzyme variant
