# Supplementary material for: Cinchonine induces apoptosis of HeLa and A549 cells through targeting TRAF6
Source: J Exp Clin Cancer Res. 2017 Feb 23;36:35. doi: 10.1186/s13046-017-0502-8 (PMC5324264; doi:10.1186/s13046-017-0502-8)
Supplement: Additional file 1: — Figure S1. Chemical structures of cinchonine and probe substituted cinchonine. Figure S2. The expression level of TRAF6. (DOC 80 kb) [file 13046_2017_502_MOESM1_ESM.doc]

**Additional file 1**

**Supplemental Materials**

*For the Article*

*Entitled*

***Cinchonine Induced Apoptosis of HeLa and A549 Cells***

***Through Targeting TRAF6***

Yonghao Qi,^1^ Ambara R. Pradipta,^2^ Miao Li,^1^ Xuan Zhao,^1^ Lulu Lu,^1^ Xuegang Fu,^1^ Jing Wei,^1^ Richard P. Hsung,^3^ Katsunori Tanaka,^2,4,5^,* and Lijun Zhou^1,6^,*

*^1^Tianjin Key Laboratory for Modern Drug Delivery & High-Efficiency,*

*School of Pharmaceutical Science and Technology, Tianjin University, Tianjin 300072, P.R. China*

*^2^Biofunctional Synthetic Chemistry Laboratory, RIKEN, 2-1 Hirosawa*

*Wako, Saitama 351-0198, Japan*

*^3^School of Pharmacy, University of Wisconsin, 777 Highland Avenue*

*Madison, WI 53705-2222, USA*

*^4^Biofunctional Chemistry Laboratory, A. Butlerov Institute of Chemistry*

*Kazan Federal University, 18 Kremlyovskaya Street, Kazan 420008, Russia*

*^5^JST-PRESTO, 2-1 Hirosawa, Wako, Saitama 351-0198, Japan*

*^6^Lead Contact*

^*^Correspondence: lijunzhou@tju.edu.cn (L.Z.) and kotzenori@riken.jp (K.T.)

**I. Chemical structures of cinchonine and probe substituted cinchonine.**

**
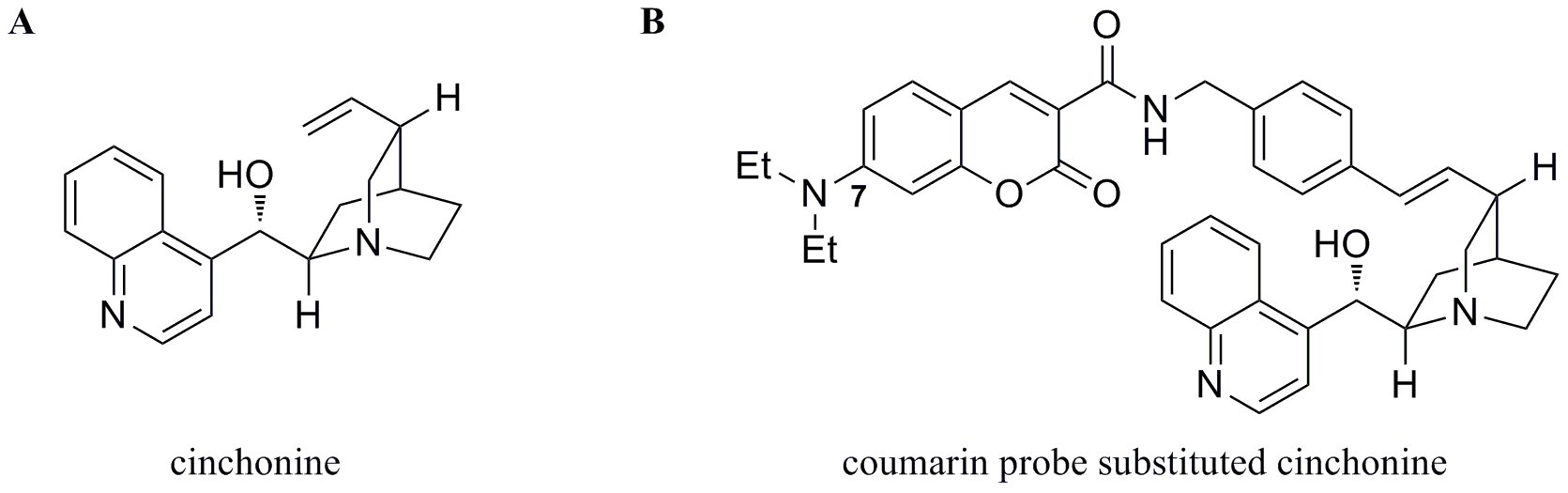
**

Figure S1. The chemical structure of (A) cinchonine, and (B) coumarin-labeled cinchonine.

**II. The expression level of TRAF6 in HeLa, A549, and NHDF cells.**

Cells were cultured in 60-mm plate. When the confluence of the cells reached 60%-70%, cells were collected. The protein were extracted with lysis buffer for 30 min on ice. Extracts were centrifuged at 12000×g for 20 min at 4 ^o^C, and the supernatants containing total protein were harvested. Each sample containing 50 μg protein was separated by 10% SDS-PAGE and transferred to PVDF membranes. The membrane was blocked in 5% non-fat milk and incubated overnight at 4 ^o^C with anti-bodies against TRAF6 (1:1000) and β-actin (1:1000). Then the membranes were incubated at room temperature for 1 h with their corresponding secondary anti-bodies. The membranes were incubated with ECL solution (CWBIO) and exposed to ECL film (FUJI, Japan). The membranes were stripped and reblotted with β-actin antibody to verify the equal loading of protein in each lane. Image J software v.1.48u (National Institutes of Health, Bethesda, MD, USA) was used to quantify the intensity of protein bands.


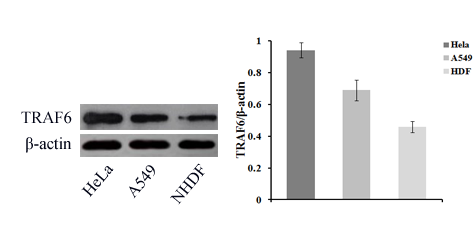


Figure S2. The expression level of TRAF6 in HeLa, A549, and NHDF cells.
