# Supplementary material for: Cinchonine induces apoptosis of HeLa and A549 cells through targeting TRAF6
Source: J Exp Clin Cancer Res. 2017 Feb 23;36:35. doi: 10.1186/s13046-017-0502-8 (PMC5324264; doi:10.1186/s13046-017-0502-8)
Supplement: Additional file 2: Table S1. — Weight of the nude mice. (DOCX 15 kb) [file 13046_2017_502_MOESM2_ESM.docx]

| Day 2 Day 4 Day 6 Day 8 Day 10 Day 12 Day 14  Day  Group |
| --- |
| 19.8 g 19.3 g 19.1 g 19.4 g 19.1 g 19.6 g 19.9 g  20.5 g 20.2 g 20.7 g 20.5 g 20.6 g 21.0 g 21.5 g  Control 18.4 g 18.9 g 18.3 g 18.4 g 19.0 g 19.0 g 19.1 g  18.0 g 18.7 g 18.7 g 19.1 g 18.5 g 18.2 g 18.9 g  18.0 g 18.3 g 18.3 g 17.7 g 18.1 g 18.5 g 18.4 g    18.9 g 19.1 g 18.9 g 19.2 g 19.1 g 19.2 g 19.2 g  17.7 g 18.0 g 18.2 g 17.4 g 18.0 g 18.2 g 18.3 g  Experiment I 19.5 g 19.2 g 19.1 g 19.6 g 19.5 g 19.9 g 19.8 g  18.2 g 18.3 g 18.4 g 18.3 g 18.5 g 18.5 g 18.8 g  18.4 g 18.6 g 18.8 g 18.4 g 19.0 g 19.1 g 19.1 g    18.1 g 18.4 g 18.4 g 18.3 g 18.1 g 18.6 g 18.5 g  20.3 g 20.6 g 20.2 g 20.3 g 20.2 g 20.6 g 21.0 g  Experiment II 18.4 g 18.9 g 18.9 g 19.1 g 19.0 g 19.0 g 19.3 g  18.5 g 18.2 g 18.3 g 18.4 g 18.3 g 18.4 g 18.7 g  18.2 g 18.2 g 18.4 g 17.9 g 18.3 g 18.3 g 18.9 g |

Table S1. Weight of the nude mice

Additional file 2
